# Statin discontinuation in persons with and without Alzheimer’s disease

**DOI:** 10.1007/s00228-022-03320-3

**Published:** 2022-04-21

**Authors:** Mai Vu, Raimo Kettunen, Anna-Maija Tolppanen, Sirpa Hartikainen, Heidi Taipale

**Affiliations:** 1 Kuopio Research Centre of Geriatric Care, University of Eastern Finland, Kuopio, Finland; 2 School of Pharmacy, University of Eastern Finland, PO Box 1627, 70210 Kuopio, Finland; 3 School of Medicine, University of Eastern Finland, Kuopio, Finland; 4 Department of Forensic Psychiatry, Niuvanniemi Hospital, University of Eastern Finland, Kuopio, Finland; 5 Department of Clinical Neuroscience, Karolinska Institutet, Stockholm, Sweden; 6 Center for Psychiatry Research, Stockholm City Council, Stockholm, Sweden

**Keywords:** Statins, Discontinuation, Alzheimer’s disease, Primary prevention, Secondary prevention, Register-based study

## Abstract

**Background:**

Although statin use is reported to decrease after dementia diagnosis, time to statin discontinuation and factors associated with discontinuation have not been studied in persons with Alzheimer’s disease (AD). We compared the risk of discontinuation and factors associated with discontinuation, including secondary and primary prevention indication, in statin users with and without AD.

**Methods:**

The register-based Medication Use and Alzheimer’s Disease (MEDALZ) cohort includes community dwellers with a clinically verified AD diagnosed during 2005–2011 in Finland. On the AD diagnosis date (index date), each person with AD was matched with a comparison person without AD. We included 25,137 people with AD and 22,692 without AD who used statin on the index date or initiated within 90 days after. Cox regression models restricted to 4-year follow-up were conducted.

**Result:**

The median time to statin discontinuation was 1.46 years in people with AD and 1.36 years in people without AD. People with AD were more likely to discontinue than people without AD (adjusted HR (aHR) 1.20 (95% CI 1.18–1.24)). This was observed for both primary (aHR 1.11 (1.06–1.16)) and secondary prevention (aHR 1.30 (1.25–1.35)) purpose. Factors associated with discontinuation included higher age and female gender, whereas concomitant cardiovascular drug use and previous statin use were associated with decreased risk.

**Conclusion:**

The absolute difference in discontinuation rates was small, and the same factors were associated with statin discontinuation in people with and without AD. The findings suggest that cognitive decline plays a minor role on statin discontinuation.

**Supplementary information:**

The online version contains supplementary material available at 10.1007/s00228-022-03320-3.

## Introduction

Statins are used for prevention of atherosclerotic cardiovascular diseases. However, there have been doubts about statin efficacy in older persons, especially when used for primary prevention [1]. Previous studies have showed that the prevalence of statin use declines after dementia/Alzheimer’s disease (AD) diagnosis [2, 3]. An Australian study of persons over 65 years with dementia observed that over a half of them (58.7%) discontinued statin use during a 3-year follow-up [4]. In a United Kingdom (UK) cohort study conducted in primary care, long-term statin users with dementia were more likely to discontinue statins than people without dementia, regardless of whether statins were prescribed for primary or secondary prevention [5]. In a Danish study of persons over 70 years, 33% long-term users with dementia discontinued statin therapy [6].

Although the more common discontinuation in people with dementia has consistently been reported, it is unknown when statins are discontinued in persons with AD and which characteristics, including primary or secondary prevention, are associated with discontinuation. Hence, the purpose of our study was to investigate the time to statin discontinuation from AD diagnosis and to compare the risk of statin discontinuation and associated factors in persons with and without AD.

## Methods and material

### The Medication Use and Alzheimer’s Disease study

Study was conducted on the MEDALZ (Medication Use and Alzheimer’s Disease) study. MEDALZ includes 70,718 community-dwelling people who got a clinically verified AD diagnosis during 2005–2011 [7] and a matched comparison cohort without AD. The Special Reimbursement Register, maintained by the Social Insurance Institution of Finland (SII), was used to identify persons with AD. This register also contains information on reimbursement according to specific chronic diseases such as diabetes, cardiovascular diseases, and Alzheimer’s disease. This study utilized data from Prescription Register, the Special Reimbursement Register, the Care Register for Health Care, and Statistics Finland.

The AD diagnosis is consistent with criteria of NINCDS–ADRDA (National Institute of Neurological and Communicative Disorders and Stroke and the Alzheimer’s Disease and Related Disorders Association) and DSM-IV criteria for AD (Diagnostic and Statistical Manual Fourth edition) [8, 9] including computed tomography or magnetic resonance, exclusion of alternative diagnosis, and confirmation of diagnosis by a geriatrician or neurologist. Hence, at the time of AD-diagnosis, the MEDALZ cohort contains persons from mild to moderate stages of AD.

Each person in the AD cohort was matched with one comparison person without AD by age (± 1 year), sex, and region of residence at the date of AD diagnosis. The comparison persons were identified from the Social Insurance Institution of Finland database including all residents. To fulfill the criteria of matching, those persons had to meet criteria including (a) alive and community-dwelling on the last day of the month when case was diagnosed with AD, and (b) no special reimbursement for AD medication or acetylcholinesterase inhibitor or memantine purchase before or within 12 months after matching date. The matching date was assigned as index date for these comparison persons.

### Identification of statin users

Statin users were identified from Prescription Register with ATC code C10AA. Statin treatment episodes were constructed by AdhereR package of R [10] which calculates duration of use based on purchase dates, assumed or prescribed daily dose (in tablets per day), and with allowed gap (grace period) defined by the investigator. We assumed that statins were used 1 tablet per day [11]. Because we do not have information of drug use during stay in hospital/nursing home, we censored statin users who had hospital stay longer than 60 days to minimize misclassification and the risk of exposure misclassification. A sensitivity analysis was performed with censoring to < 30-day stays.

This study included people who had a treatment episode ongoing on the date of AD diagnosis or the corresponding (index date for persons without AD) or who initiated statin use day within 90 days after the index date. (Supplementary Fig. 1).

Cohort entry date was defined as the date AD diagnosis or the first date of the first statin purchase that began within 90 days after the index date.

### Statin discontinuation

Discontinuation was defined as not filling a statin prescription during the days’ supply of the previous dispensing plus grace period. Grace period is an allowed gap which is added to the drug use duration to deal with non-perfect adherence and other variation in purchasing behavior. We used a 120-day grace period to capture true discontinuation. In Finland, medication can be dispensed for 90 days (maximum of 100 days) of treatment at the time and grace period reflects this.

The maximum follow-up time was restricted to 4 years to ensure all people in cohort had the same possible observation period. People were followed up until discontinuation of statin use, and censored to death, over 60 days of hospitalization, end of follow-up (4 years or December 31, 2015) or date when persons without AD were diagnosed with AD, whichever came first.

### Covariates

Statin use was categorized as for primary and secondary prevention. Secondary prevention was defined using ICD-10 codes for cardiovascular (CV) diseases including coronary artery disease, coronary artery bypass grafting (CABG) or percutaneous coronary intervention (PCI), ischemic strokes, and atherosclerosis of any neck or brain arteries. Primary prevention was defined as having no conditions defined as secondary prevention. Diagnosis codes, data sources, and time periods are defined in Supplementary Table 1.

Data on comorbidities (chronic heart failure, atrial fibrillation, and diabetes) were extracted from the Special Reimbursement and Care Register for Health Care (including hospital discharges and specialized healthcare outpatient visit) based on ICD 10 (Supplementary Table 2).

A number of cardiovascular drug substances other than statins were extracted from the Prescription Register data with ATC code C* excluding C01C, C04, C05, C10AA. The prevalence of cardiovascular drug substances was identified in 120-day period before and after the index date because this time window matched with our grace period definition and should capture all regular users. The number of cardiovascular drugs substances used was calculated by taking into account on the actual number of drug substances from combination products.

### Statistical analysis

We performed descriptive statistics as means, standard deviations (SD), median (interquartile range (IQR)), or frequency and percentages where appropriate. We applied *T* test for continuous variables with normal distribution, Mann–Whitney *U* test for continuous variables with skewed distribution, and chi-square test for categorizing variable to compare characteristics between groups. We presented results with 95% confidence intervals.

We used Cox regression models to compare the risk of statin discontinuation between people with and without AD and to assess factors related to statin discontinuation in persons with and without AD. The results were adjusted for age at cohort entry, sex, statin use before cohort entry, indication, sum of cardiovascular drug substances, diabetes, atrial fibrillation, heart failure, calendar year, hospital district. The proportionality assumption was confirmed with Kaplan–Meier curves.

To assess factors associated with statin discontinuation, the same analyses were performed between persons with and without AD and stratified based on primary/secondary prevention and AD status.

To evaluate whether the results were affected by choice of grace period and maximum allowed length of hospital stay, sensitivity analyses were performed with grace period of 90 days and by censoring to 30-day hospital stays.

To illustrate temporal trends in the cohort, the proportions of statin users of each annual cohort (per AD diagnosis year), as well as the cumulative survival (i.e., continuation of statin use), are presented as supplementary analyses.

All statistical analyses were performed using the software R 4.0 program [12] and STATA 14 (Stata Corporation, College Station, TX, USA).

## Results

### Characteristics of statin users in both cohorts

Altogether, 25,137 persons with AD and 22,692 persons without AD used statin on the index date (date of AD diagnosis) or initiated statin within 90 days after the index date (Table 1). The mean age on cohort entry was approximately 79 years, and majority of persons in both cohorts were women. Statin users with AD had higher prevalence of diabetes, atrial fibrillation, and chronic heart failure than persons without AD.

**Table 1:** Characteristics of statin users with Alzheimer’s disease (AD) and non-AD cohorts at the date cohort entry

	AD (*n* = 25,137)	No AD (*n* = 22,692)	*p* value
**Age in years at cohort entry (mean, SD)**	79.1 (6.3)	79.3 (6.1)	< 0.0001
< 70	1700 (6.8)	1386 (6.1)	< 0.0001
70–74	3582 (14.2)	3053 (13.4)	
75–79	7011 (27.9)	6268 (27.6)	
80–84	8028 (31.9)	7596 (33.5)	
≥ 85	4816 (19.1)	4389 (19.3)	
Sex (women) (*n*,%)	16,003 (63.7)	14,478 (63.8)	0.752
**Highest occupational social class before AD**			< 0.0001
Managerial/professional	5424 (21.6)	5169 (22.8)	
Office	2146 (8.5)	1984 (8.7)	
Farming/forestry	4794 (19.1)	4502 (19.8)	
Sales/industry/cleaning	10,956 (43.6)	9353 (41.2)	
Unknown	1817 (7.2)	1684 (7.5)	
Median (IQR) follow-up time (years)	2.72 (1.1–4.0)	4.0 (1.6–4.0)	
**Reason for end of follow-up**			< 0.0001
Statin discontinuation	9931 (39.5)	7880 (34.7)	
End of follow-up	8749 (34.8)	11,663 (51.4)	
Over 60 days hospitalization	4026 (16.0)	985 (4.3)	
Death	2431 (9.7)	1713 (7.6)	
Non AD diagnosed with AD	0	451 (2)	
Median (IQR) time to discontinuation (years)	1.46 (0.5–2.5)	1.36 (0.5–2.6)	
***Duration of statin use before cohort entry (years)***			< 0.0001
0	1977 (7.9)	1131 (5.0)	
1–3	9653 (38.4)	7677 (33.8)	
4–6	5,095 (20.3)	5,338 (23.5)	
7–10	5,038 (20.0)	5,233 (23.1)	
Over 10	3,374 (13.4)	3,313 (14.6)	
***Comorbidities***			
Diabetes	6892 (27.4)	5489 (24.2)	< 0.0001
Atrial fibrillation	4831 (19.2)	3964 (17.5)	< 0.0001
Chronic heart failure	4141 (16.5)	3510 (15.5)	0.003
***Primary/secondary prevention***			< 0.561
Primary prevention	11,369 (45.2)	10,203 (45.0)	
Secondary prevention	13,768 (54.7)	12,489 (55.0)	
Ischemic coronary artery diseases and PCI/CABG	12,006 (47.8)	11,135 (49.1)	
Ischemic strokes, atherosclerosis of neck and brain arteries	3,648 (14.5)	2,839 (12.5)	
***Number of other cardiovascur drug substances than statin***			< 0.0001
0	3888 (15.5)	2765 (12.2)	
1–2	10,734 (42.7)	8974 (39.5)	
3–4	8266 (32.9)	8365 (36.9)	
5 and more	2249 (8.9)	2588 (11.4)	
***Number of statin users in calendar year***			0.323
2005	2303 (9.2)	2120 (9.3)	
2006	2555 (10.2)	2434 (10.7)	
2007	3110 (12.4)	2867 (12.6)	
2008	3645 (14.5)	3271 (14.4)	
2009	4128 (16.4)	3700 (16.3)	
2010	4437 (17.6)	3934 (17.3)	
2011	4959 (19.7)	4366 (19.4)	

During the 4-year follow-up, 39.5% of people with AD and 34.7% in of those without AD discontinued statin use (Table 1). The median time from the cohort entry date to the date of discontinuation was similar in both cohorts: 1.46 years in persons with AD and 1.36 years in persons without AD. In both cohorts, over half of persons (55%) had a secondary prevention indication for statin use. Over 90% of people used statins already before cohort entry and over 80% used at least one other cardiovascular drug.

### Rates of statin discontinuation

The rate of statin discontinuation was 4.35/10000 person-years in persons with AD and 3.28/10000 person-year in those without AD (Table 2). The relative risk for discontinuation in people with AD was 20% higher than in people without AD (adjusted hazard ratio (aHR) 1.20, 95% CI 1.18–1.24). The relative difference between AD and comparison group was slightly larger in secondary prevention (aHR 1.30, 95% CI 1.25–1.35) than in primary prevention (aHR 1.11, 95% CI 1.06–1.16).

**Table 2:** Association of Alzheimer’s disease with statin discontinuation

		AD			No AD			HR	
		Number of observation	Number of events	Event/10000 person-years	Number of observation	Number of events	Event/10000 person-years	Unadjusted	Adjusted
***Main analysis***									
Grace period 120 days and censoring to > 60 days hospital care	Total	25,137	9931	4.35	22,692	7880	3.28	1.31 (1.27–1.35)	1.20 (1.18–1.24)
	Primary	11,065	4633	4.44	9917	3736	3.55	1.25 (1.20–1.31)	1.11 (1.06–1.16)
	Secondary	14,072	5298	4.27	12,775	4144	3.08	1.39 (1.31–1.43)	1.30 (1.25–1.35)
***Sensitivity analyses***									
Grace period 120 days and censoring to > 30 days hospital care	Total	25,124	9005	4.09	22,692	7568	3.19	1.26 (1.23–1.30)	1.15 (1.12–1.19)
	Primary	11,368	4400	4.26	10,203	3727	3.47	1.21 (1.16–1.26)	1.06 (1.02–1.11)
	Secondary	13,756	4605	3.94	12,489	3841	2.97	1.32 (1.26–1.37)	1.25 (1.19–1.30)
Grace period 90 days and censoring to > 60 days hospital care	Total	26,171	7954	3.03	23,931	4773	1.66	1.85 (1.79–1.93)	1.81 (1.77–1.88)
	Primary	11,921	3777	3.03	10,918	2258	1.67	1.83 (1.73–1.92)	1.75 (1.66–1.84)
	Secondary	14,250	4177	3.04	13,013	2515	1.64	1.89 (1.80–1.98)	1.88 (1.79–1.97)

Adjusted: age at cohort entry, sex, statin use before cohort entry, sum of cardiovascular drug substances, hospital district, diabetes, atrial fibrillation, heart failure, calendar year

Reference group is no AD

In sensitivity analyses with censoring to 30-day hospitalization, the difference between people with and without AD was similar to that in the main analyses (Table 2). In sensitive analyses with shorter (90 days) grace period, the difference between people with and without AD became larger (80% higher relative risk).

### Factors associated with statin discontinuation between people with and without AD

The same characteristics were associated with statin discontinuation in people with and without AD (Table 3). Discontinuation was less common in men than in women and most common in age groups over 75 years or over in people with AD and in age groups 70 years or over in people without AD. The risk of discontinuation was not different between primary and secondary prevention indication users, or those with and without diabetes or atrial fibrillation (Table 3). Heart failure was weakly associated with risk of discontinuation. The risk of discontinuation was lower among users who used higher number of other cardiovascular drugs, or who had used statins before the cohort entry. The association between duration of previous statin use was stronger in people without AD. In both cohorts, statin discontinuation was more common in those with later cohort entry years in comparison to those with index date in 2005 (Table 3, and Supplementary Fig. 2).

**Table 3:** Factors associated with statin discontinuation stratified by AD status

	AD		No AD	
	**Unadjusted HR**	**Adjusted HR**	**Unadjusted HR**	**Adjusted HR**
**Age at cohort entry**				
< 70 years	ref	ref	ref	ref
70–74	1.06 (0.97–1.17)	1.11 (1.00–1.22)	1.13 (1.01–1.27)	1.25 (1.11–1.39)
75–79	1.09 (1.00–1.20)	1.17 (1.07–1.28)	1.10 (0.99–1.22)	1.27 (1.14–1.41)
80–84	1.32 (1.21–1.44)	1.40 (1.28–1.53)	1.25 (1.13–1.38)	1.45 (1.31–1.61)
85–100	1.85 (1.68–2.02)	1.91 (1.74–2.10)	1.55 (1.40–1.73)	1.74 (1.56–1.93)
**Sex**				
Female	ref	ref	ref	ref
Male	0.88 (0.85–0.93)	0.91 (0.87–0.96)	0.87 (0.83–0.91)	0.86 (0.82–0.90)
**Indication**				
Primary prevention	ref	ref	ref	ref
Secondary prevention	0.95 (0.92–0.99)	1.00(0.96–1.04)	0.87 (0.83–0.91)	0.96 (0.92–1.01)
**Comorbidities**				
Diabetes	0.92 (0.88–0.96)	0.96 (0.92–1.01)	0.91 (0.86–0.96)	0.96 (0.91–1.02)
Heart failure	1.11 (1.05–1.17)	1.12 (1.06–1.19)	1.04 (0.98–1.11)	1.10 (1.03–1.18)
Atrial fibrillation	1.05 (1.00–1.11)	1.04 (0.98–1.09)	0.98 (0.93–1.04)	1.01 (0.95–1.08)
**Number of cardiovascular drug substance other than statin use**				
0	ref	ref	ref	ref
1–2	0.84 (0.80–0.89)	0.82 (0.78–0.87)	0.84 (0.78–0.90)	0.83 (0.77–0.89)
3–4	0.81 (0.76–0.86)	0.76 (0.72–0.81)	0.73 (0.68–0.78)	0.71 (0.66–0.76)
more than 5	0.81 (0.74–0.87)	0.74 (0.68–0.82)	0.70 (0.64–0.77)	0.67 (0.60–0.74)
**Statin use before cohort entry (years)**				
0	ref	ref	ref	ref
1–3	0.79 (0.74–0.85)	0.79 (0.74–0.85)	0.54 (0.49–0.58)	0.53 (0.48–0.57)
4–6	0.63 (0.58–0.68)	0.62 (0.58–0.68)	0.30 (0.27–0.33)	0.29 (0.26–0.32)
7–10	.058 (0.54–0.63)	0.57 (0.53–0.62)	0.25 (0.23–0.27)	0.23 (0.21–0.26)
Over 10	0.61 (0.56–0.67)	0.56 (0.51–0.61)	0.26 (0.23–0.29)	0.22 (0.20–0.25)
**Calendar year**				
2005	ref	ref	ref	ref
2006	0.98 (0.89–1.08)	0.98 (0.89–1.09)	0.95 (0.85–1.06)	0.97 (0.88–1.09)
2007	1.04 (0.95–1.15)	1.06 (0.97–1.17)	1.00 (0.91–1.11)	1.05 (0.95–1.16)
2008	1.15 (1.05–1.26)	1.16 (1.06–1.27)	1.18 (1.06–1.30)	1.29 (1.17–1.43)
2009	1.29 (1.18–1.41)	1.31 (1.21–1.43)	1.29 (1.17–1.41)	1.46 (1.33–1.60)
2010	1.48 (1.36–1.61)	1.51 (1.38–1.64)	1.48 (1.35–1.63)	1.71 (1.56–1.88)
2011	1.43 (1.32–1.55)	1.46 (1.35–1.59)	1.44 (1.32–1.58)	1.71 (1.56–1.88)

Adjusted by: age at cohort entry, sex, statin use before cohort entry, indication, sum of cardiovascular drug substances, diabetes, atrial fibrillation, heart failure, calendar year, hospital district

## Discussion

To our knowledge, this is the first study comparing the statin discontinuation risk and factors associated with discontinuation between people with and without AD. Although people with AD had higher relative risk of statin discontinuation than people without AD during the 4-year follow-up, the absolute difference in discontinuation rates was small. The same factors were associated with discontinuation in people with and without AD, as older people and women were more likely to discontinue whereas users of other cardiovascular drugs and those who had used statins for longer time before the index date were less likely to discontinue in both cohorts. Discontinuation was equally common in primary and secondary indication in both cohorts.

Most of the previous studies on statin discontinuation in people with dementia have applied new user design [4, 5, 13] and only one study included prevalent long-term statin users [6]. The proportion of people with dementia who discontinued in these previous studies has ranged between 33 [6] and 59% [4], with the smallest proportion being observed in the study of long-term statin users. The proportion of people who discontinued in our study is comparable with the earlier Danish study of long-term users [6], although direct comparisons are not meaningful due to differences in study design. Our study included both prevalent users and those who initiated within 90 days of AD diagnosis, with the majority being prevalent users. In the previous studies, discontinuation was more common in people with dementia in the study with long-term statin users [6] while the opposite association was observed in studies conducted among incident short-term users [5, 13].

In our study, statin discontinuation was relatively common in both cohorts and the highest HRs were observed in the oldest age groups. This finding is in line with previous studies [6, 13, 14]. In a Danish study, the difference in the discontinuation between age groups 70–74 years and those aged > 95 years was twofold (aOR = 2.06, 95% CI 1.35–3.16) at 1 year and nearly fourfold at 4 years (aOR3.94, 95% CI 1.83–8.49) of follow-up [13]. A third of participants in our study were aged over 80 years and nearly fifth were at least 85 years old which may partly explain the same kind of risk of discontinuation in both cohorts. In addition, the oldest persons are at the higher risk of statin-related adverse effects [15], and the health status is more unstable than in younger persons, which may explain the higher discontinuation rates among the older participants in our and earlier studies. The changes in health status may also have led to deprescribing. Other comorbidities or progression of disease which negatively affect life expectancy such as cancer [16] could also affect decision of statin deprescribing [17]. In addition, frailty, which is common among older persons with high age and even more common in persons with AD [18], might increase the decision to deprescribe [19]. Although regular medication reviews are recommended by the Finnish authorities, those recommendations are not always applied in clinical practice; therefore, we do not expect that statin discontinuation rates in our study were significantly affected by regular reviews.

Time period–related trends have been observed in the use of statins among older persons aged over 79 years in USA that the proportion of statin users increased from the year 1999 until 2009 in secondary prevention and until 2007 in primary prevention, then decreased after that [20]. Similarly, there was an increase in prevalence of statin use between years 2008 and 2010 among people aged 65 years in Finland and prevalence remained at the same level after that to the end of year 2015 [21]. Consistent with this, we observed an increase in the prevalence of statin use per diagnosis year until 2011, while the discontinuation rate was also higher in those who entered the cohort in later years. Public discussion on whether statins should be used for other than secondary prevention indications [22] and relatively high drug prices but low reimbursement together with time trends described may have impacted our study results. Only a small proportion of statin users in Finland reported to have discontinued statin therapy due to worrying or experienced of side effects [23].

Our results showed that discontinuation was not different due to primary versus secondary prevention in both people with and without AD, which is in line with Danish study [6]. It could be due to consideration of clinicians in the period when benefits of statin in primary prevention were still debated [24]. However, the discontinuation risk was lower among users of other cardiovascular drugs. It could be partly because we may not have captured all milder cardiovascular diseases and consequently in the primary prevention group may include persons with mild coronary disease or atherosclerosis of other arteries. Therefore, it is possible that the number of other cardiovascular drugs better describes the presence and severity of cardiovascular diseases than our diagnosis-based measure of secondary versus primary prevention. Besides, using statin in long period increases adherence to statin use which partially explained the necessaries of statin therapy and stronger commit to therapy in these cases [25].

The slightly higher risk of discontinuation in persons with AD in our study, observed in both primary and secondary prevention indication, may also be due to lower adherence in persons with cognitive decline [26–28] that is also reported in Finland [29]. However, for persons with dementia, caregiver or home care services often take care of medications instead of the patient and in this kind of situation, adherence does not describe the behavior of the patient. The relative risk of statin discontinuation in our study is comparable to findings from a systematic review that reported 18% higher risk of statin discontinuation in persons with dementia compared to those without dementia [14].

### Strengths and limitations

Use and linkage of different registers from a country with public healthcare system allowed us to perform a nationwide study with low risk of selection bias. We used Prescription Register which captures dispensed medication; thus, the time people redeemed prescription was more precise than prescriptions. Moreover, we use ATC code C10AA which captured statins in general thus accounting for switches into another statin.

However, our study has some limitations. Firstly, this is a register-based study; thus, like other studies, we do not know whether discontinuation decision is made by prescriber (i.e., deprescribing) or by the caregiver or patient and we do not know whether medications were actually taken by patients. We also lack information about changes in severity of comorbidities during the follow-up, which could impact on discontinuation of statin therapy in both cohorts. We also lacked data on patient-related factors such as income that has been previously linked to discontinuation [30]. Due to lacking indication in register data, we may have misclassified some people into primary prevention category. Therefore, further research is needed on the reasons of statin discontinuation, as well as by who and how the decision of statin discontinuation is initiated and made.

## Conclusion

Discontinuation was common in both groups and the absolute difference in statin discontinuation rates was small between people with and without AD. These findings suggest that cognitive decline does not have a large impact on discontinuation of statins in older persons.

## Supplementary Information

Below is the link to the electronic supplementary material.

Supplementary file1 (DOCX 376 kb)

## Data Availability

The authors are unable to openly share the data used to conduct this study. The data that support the findings of this study are available from the corresponding author upon reasonable request and with permission of the register maintainers.
