# Supplementary material for: Statin discontinuation in persons with and without Alzheimer’s disease
Source: Eur J Clin Pharmacol. 2022 Apr 21;78(7):1145–53. doi: 10.1007/s00228-022-03320-3 (PMC9184400; doi:10.1007/s00228-022-03320-3)
Supplement: Supplementary file 1 — Supplementary file1 (DOCX 376 kb) [file 228_2022_3320_MOESM1_ESM.docx]

**Supplementary material**

**Supplementary table 1:** Code were used to identify secondary prevention

| Variable | Data sources and coding | Time |
| --- | --- | --- |
| CABG | Care register for health care NOMESCO FNA, FNC, FNE, code AA in the extra sheet of cardiac patient  Care register for health care**:** ICD-10 Z95 | From 1996 to cohort entry |
| PCI | Care register for health care NOMESCO FNG00, FNG10, FN1AT, FN1BT, FN1YT, FN2, FN_2 , codes AN2, AN3, AN4 in the extra sheet of cardiac patient  Care register for health care**:** ICD-10 Z95 | From 1996 to cohort entry |
| Coronary artery disease | Special reimbursement register code 206, 213, 280  Care register for health care ICD-10 I20-I25 | From 1996 to cohort entry |
| Atherosclerosis of all arteries of neck and brain including ischemic strokes | Care register for health care: ICD-10 I63-66 | From 1996 to cohort entry |

**Supplementary table 2**: Code were used to identify comorbidities

| Variable | Data sources and coding | Time |
| --- | --- | --- |
| Atrial fibrillation | Care register for health care ICD 10-I48 | From 1996 to cohort entry |
| Chronic heart failure | Special reimbursement register code 201  Care register for health care ICD-10 I42, I43, I50, I110 | From 1996 to cohort entry |
| Diabetes | Prescription register: ATC code A10 excluding A10BX01(guar gum)  Special reimbursement register code 103 | From 1996 to cohort entry |
| Cardiovascular substance other than statin | Prescription register: ATC code C* excluding | Before and after index date (date AD diagnosis) 120 days |


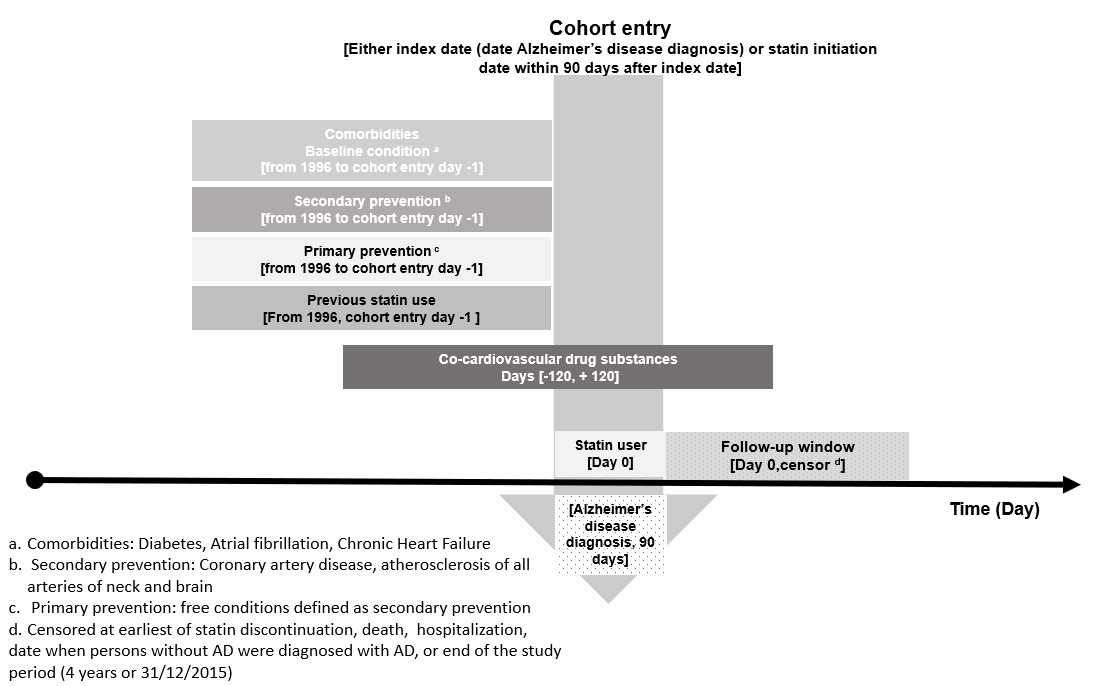


**Supplement figure 1**: Study design


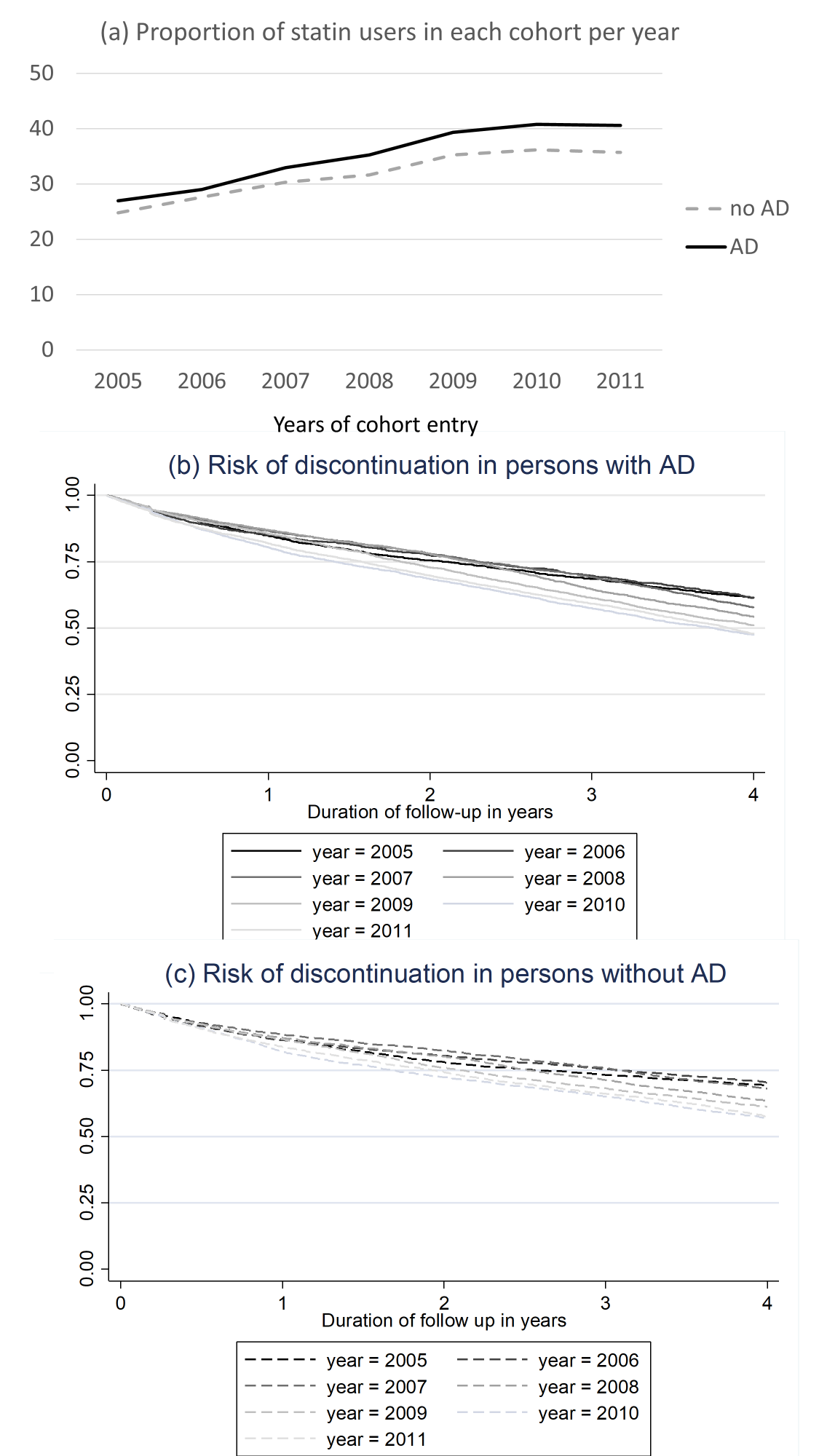


**Supplement figure 2:** Proportion of statin users in people with and without AD (a) and risk of discontinuation in people with AD (b) and people without AD (c) in study time 2005 – 2011.
